# Underutilization of coper/non-coper screening in anterior cruciate ligament injuries management in Italy: an online survey

**DOI:** 10.3389/fresc.2024.1497828

**Published:** 2025-01-15

**Authors:** Luca Macrelli, Luca Mallia, Gabriele Thiebat, Jacopo Emanuele Rocchi, Lee Herrington, Sebastiano Nutarelli

**Affiliations:** ^1^ Musculoskeletal Physical Therapy, University of Bologna, Bologna, Italy; ^2^ Department of Movement, Human and Health Sciences, Foro Italico University of Rome, Rome, Italy; ^3^ Sport Traumatology and Minimally Invasive Surgery Center, IRCCS Istituto Ortopedico Galeazzi, Milan, Italy; ^4^ FIFA Medical Centre of Excellence, Villa Stuart Sport Clinic, Rome, Italy; ^5^ Manchester Institute for Health and Performance, English Institute of Sport, Manchester, United Kingdom; ^6^ Centre for Health, Sport and Rehabilitation Sciences, University of Salford, Salford, United Kingdom; ^7^ Department of Surgery, EOC, Service of Orthopaedics and Traumatology, Lugano, Switzerland; ^8^ School of Public Health, Physiotherapy and Sports Science, University College Dublin, Dublin, Ireland

**Keywords:** ACL deficient, rehabilitation, non-surgical, orthopedic surgeon, physiotherapist

## Abstract

**Introduction:**

Total and sub-total lesions of the anterior cruciate ligament (ACL) are one of the most frequent and performance-limiting injuries to the knee joint within the active population. Early surgical management, often regarded as the primary management strategy, has recently been shown to have similar outcomes when compared with an initial rehabilitative approach followed by surgical ACL reconstruction if higher levels of functionality are needed. The primary objective of the study was to investigate the physiotherapists and orthopedic surgeons’ “coper/non-coper” screening application in the clinical management of the patient after ACL injury. Second, the authors aimed to investigate the cooperation between physiotherapists and orthopedic surgeons when dealing with patients with ACL injuries.

**Methods:**

An online survey consisting of 12 questions on the clinical practice of the surveyed physiotherapists (*n* = 803) and orthopedic surgeons (*n* = 201), and the relation between these healthcare professionals, was distributed. The answers were stratified depending on clinical experience in dealing with ACL injuries.

**Results:**

Both physiotherapists and orthopedic surgeons showed a low degree of confidence and application of the “coper/non-coper” screening when managing ACL lesions. The sub-population of ACL experts reported a good level of interprofessional interaction. Nevertheless, an updated level of knowledge regarding the recent evidence on the non-surgical management of ACL lesions is still lacking.

**Conclusions:**

The study findings indicate the need to improve the collaboration between Italian physiotherapists and orthopedic surgeons as well as their knowledge of the non-surgical approaches to ACL lesions.

## 1 Introduction

Injuries of the anterior cruciate ligament (ACL) are one of the most frequent and performance-limiting injuries to the knee within the active population. In the USA, ACL injuries exceed 200,000 cases per year (1). Early surgical management is regarded as the primary management strategy but has recently been shown to have similar outcomes when compared with an initial rehabilitative approach and optional delayed ACL reconstruction (ACL-R) (2–4).

After an isolated ACL lesion, some individuals can avoid ACL-R while returning to pre-injury functional levels through an exercise rehabilitation intervention, while others continue to report dynamic instability of the knee despite undergoing rehabilitation. Members of the former group, able to return to function at a high level in level I sports (5) at least weekly after injury without complaint of instability, are defined as “copers,” while those in the latter group are labeled as “non-copers” (6).

The “coper/non-coper screening” (CNCS) concept dates back to the 2000s when Fitzgerald et al. (7) used a cluster of tests and questionnaires to identify individuals who could postpone and possibly avoid surgery based on their functionality (7). A 2008 study showed that 70% of individuals with isolated ACL lesions and initially classified as non-copers, can achieve coper status within 1 year (8).

Eitzen et al. (9) reported that a 5-week neuromuscular rehabilitation program was effective in ensuring better results for up to 2 years before ACL-R. The choice not to rush into surgery should be considered as routine for adequately profiled patients. Whether delaying surgery results in identifying actual copers or in a preoperative physical therapy period for non-copers, it was proven to result in better functional outcomes in the short term and to achieve overall better results 2 years after an eventual ACL-R (10).

Thoma et al. presented a coper/non-coper functional classification in the acute phase for individuals with ACL injuries. An evaluation performed after 5 weeks of neuromuscular physical therapy led to almost half of the initial non-copers to adapt, switching their status to copers (11). In addition, a further study reported that 28% of the professional handball players who suffered a total ACL injury were managed with a non-surgical approach, resulting in 82% of them being able to return to their pre-injury levels and remain there during a 4-year follow-up (12). Interestingly, the appearance of ACL healing after an ACL rupture occurred in one in three adult copers who decided to avoid surgery, and the healing could facilitate better clinical outcomes (13).

This practice for screening is not universal; this study intends to establish its use by Italian physical therapists (PTs) and orthopedic surgeons (OSs). Although previous surveys on ACL rehabilitation have been published (14–18), to the authors’ knowledge, the current work is the first investigating the utilization of the CNCS involving two groups of health professionals.

The underpinning hypothesis is that Italian clinicians are currently not implementing the CNCS in the management of patients with ACL injuries.

The aim of this project was to investigate the current implementation level of the CNCS (11) in clinical practice in Italy and to identify whether a collaborative decision-making approach is taken when managing ACL injuries.

## 2 Methods

### 2.1 Study design

This survey was created to investigate the clinical practice of the two most involved health professionals in the management of ACL lesions: the PTs and the OSs. The entire process was carried out following the AAPOR Survey Disclosure Checklist (19).

The authors of this study, both PTs and OSs, provided their expertise formulating a short but comprehensive set of questions. The first version of the questionnaire was drafted by two PTs (LMac, SN) and one OS (GT), reviewed by a third PT (LH), and eventually revised by a qualitative research expert to ensure consistency of the linguistic form and minimize the risk of potentially misunderstood expressions. After the standardization of the answers’ scales, the final version was eventually released online (Supplementary Material 1).

The survey comprised 12 questions regarding different aspects of the clinical practice of clinicians dealing with patients with ACL injuries, including the application of the CNCS. The answering health professionals’ experience in the ACL field (questions 1 and 2) and their geographical localization (question 3) was investigated. Their opinion on the percentage of patients who could possibly return to sport (RTS) with and without changes of direction (CoD) with no ACL-R after an ACL injury was examined (questions 4 and 5). Their familiarity level with the CNCS and whether the screening was part of their current clinical practice was deepened (questions 6 and 7). The survey examined the PTs’ level of involvement, by the OSs, in the decision-making process eventually leading to an ACL-R or a non-surgical approach (question 8). It also investigated the regular mutual collaboration between the OSs and trusted PTs (question 9) and the percentage of patients classified as copers who eventually underwent ACL-R after a first non-surgical approach (question 10). In the end, the survey enquired about the routine presence of either the PTs in the operative room and/or the OSs in the rehabilitation gym (questions 11 and 12).

### 2.2 Participants

The survey population comprised PTs and OSs practicing in Italy. Once the data collected from PTs and OSs working abroad at the time of compilation were discarded as well as the data provided by individuals who incorrectly filled out the questionnaire multiple times, the survey data were processed. This study was approved by the Ethics Committee of the University of Rome (Foro Italico) on 8 October 2021. Before taking part in the study, all the surveyed health professionals read and electronically signed the informed consent, which assured their anonymity.

### 2.3 Procedures

Both the PT and OS surveys were placed online on the “Google Forms” platform. The survey was distributed online using various methods: (1) via a dedicated newsletter from the Italian PT Association (AIFI), which resulted in 9,617 PTs opening the mail out of the 23,700 email addresses reached (40%); (2) through dedicated health professional groups/pages on popular social networks (Facebook, Instagram, and LinkedIn); and (3) by sending email invitations to both OSs and PTs (convenience sample).

The survey commenced on 10 October 2021 and ended on 1 August 2022. The analysis of the collected answers was completed on 10 August 2022.

### 2.4 Statistical analysis

After the survey closure, the data were imported to Microsoft Excel (Microsoft Corp., Redmond, WA, USA) and analyzed by one researcher (LMac). After an initial analysis of the participants geo-localization (question 3), the first step was to determine how many responding PTs and OSs had sufficient familiarity with the CNCS (minimum value arbitrarily set by the authors at 60 out of 100) and what proportion of them applied it routinely in case of ACL injury (questions 6 and 7).

The health professionals’ opinion about the feasibility of a RTS (either with or without CoD) of individuals with non-operated ACL injuries was analyzed (questions 4 and 5) as well as how many OSs cooperate with one or more trusted PTs, and vice versa (question 9).

The PTs’ and OSs’ responses were stratified, including the replies provided by health professionals who regularly treat ACL lesions using a non-surgical approach (using questions 1 and 2).

The authors used the findings from a study by Schairer et al. in 2017 to define when an OS can reasonably be considered an “expert” in the surgical treatment of ACL tears, with the conclusions indicating a minimum number of 35 ACL-Rs per year (20). In the absence of similar data for PTs in the published literature, the authors opted for the same cutoff. This reference point was used to determine the ACL-expert surveyed population. The results of this sub-analysis are presented in Section 3.3.

The answers to questions 4–7 concerning RTS, the degree of familiarity, and the effective application of the CNCS, respectively, were then assessed, dividing the answers into two groups: the surveyed ACL experts and the remaining PTs/OSs. The CNCS was defined in the survey as an initial screening followed by a rehabilitative approach lasting at least 5 weeks before proceeding with a second screening supporting the choice between ACL-R versus the continuation of a non-surgical approach (11).

Subsequently, the answers to questions 8, 10, 11, and 12 were stratified and compared between the PTs’ and OSs’ versions to investigate the actual level of collaboration between the two health professionals.

Finally, the percentages of patients who reportedly had to undergo to an ACL-R after the CNCS process were quantified (question 9) among the clinicians actually utilizing the screening process.

## 3 Results

### 3.1 Demographic analysis

The surveyed population comprised 803 PTs and 201 OSs. Within the interviewed PT population, 530 (66%) PTs worked in Northern Italy, 152 (19%) in the center of the country, and 121 (15%) in the South.

Of the interviewed rehabilitators, 597 (75%) were aged under 35 years, 171 (21%) aged 35–50 years, and 35 (4%) were older than 50 years.

Of the surveyed OSs, 148 (74%) reported to practice in the north of the country, 25 (12%) in the center, and 28 (14%) in southern Italy.

Of the compilers, 43 (22%) were aged less than 35 years, 109 (54%) were aged 35–50 years, and 49 (24%) were older than 50 years.

### 3.2 General population analysis

Of the 803 interviewed PTs, 205 (25.5%) were confident in the implementation of the CNCS, whereas the remaining 598 (74.5%) rated their confidence as insufficient, with a score of less than 60 on a scale of 0–100. In total, 251 PTs (31%) claimed to apply it, while 552 (69%) do not use the screening in their daily clinical practice. Only 137 (17%) PTs use it in their routine clinical practice, leading to poor familiarity.

**Figure 1:**
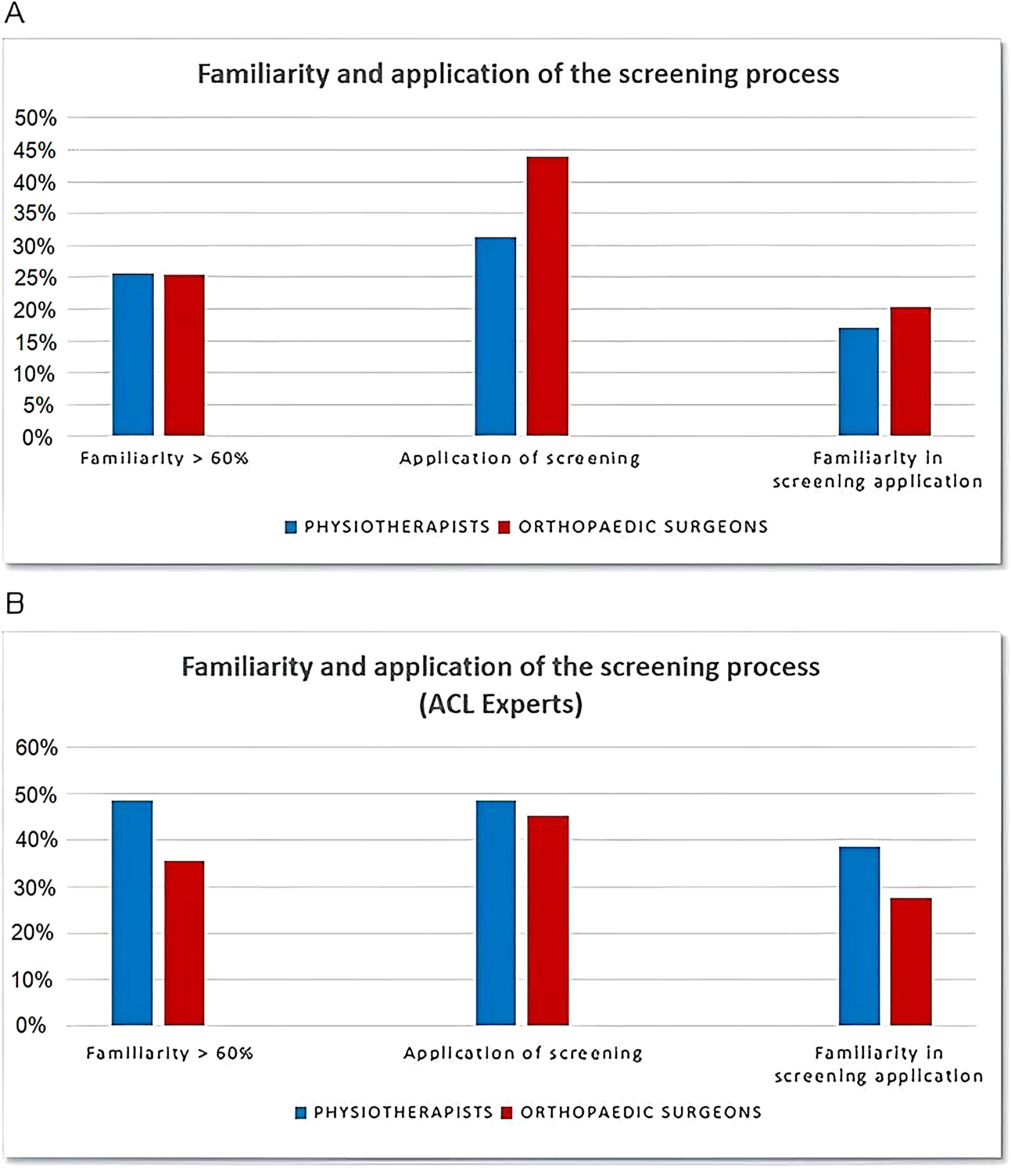
Familiarity and application of the CNCS process. **(A)** Familiarity and application of the CNCS process in the surveyed clinicians. **(B)** Familiarity and application of the CNCS process in the surveyed ACL-expert clinicians. ACL, anterior cruciate ligament; CNCS, coper/non-coper screening.

Of the 201 OSs who took part in the study, 51 (25%) replied that they felt sufficiently familiar with CNCS whereas 150 (75%) rated their confidence as less than 60 on the scale of 0–100. In total, 88 (44%) OSs refer to it whereas 113 (56%) do not utilize the screening in their everyday clinical practice. Among the physicians referring to the screening, only 41 (20%) regularly implement it, reporting a sufficient familiarity (Figure 1A).

**Figure 2:**
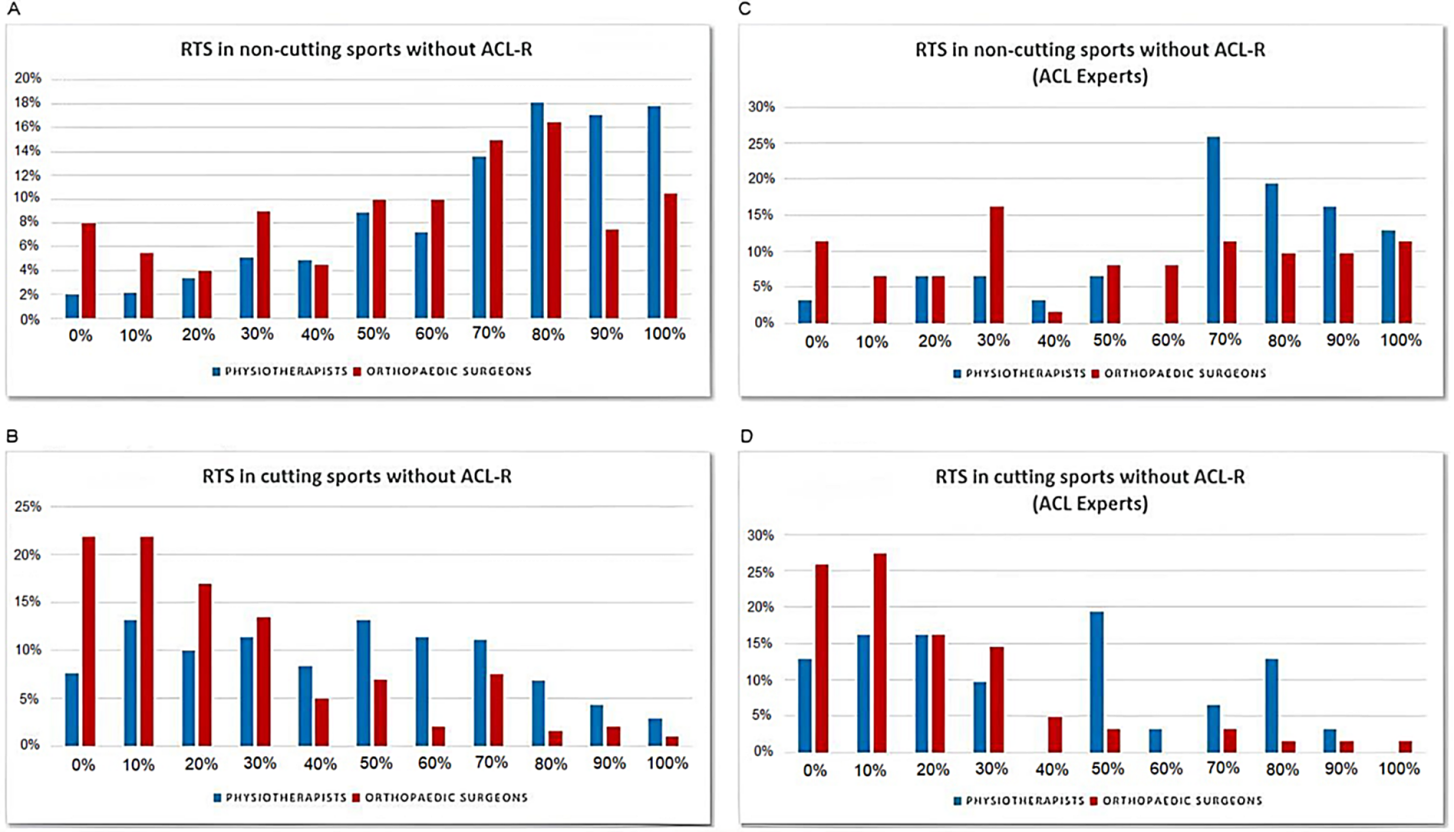
Response distribution in relation to the possibility to RTS. **(A)** Response distribution in relation to the possibility to RTS in sports without CoD (general population). **(B)** Response distribution in relation to the possibility to RTS in sports with CoD (general population). **(C)** ACL-experts’ response distribution in relation to the possibility to RTS in sports without CoD. **(D)** ACL-experts’ response distribution in relation to the possibility to RTS in sports without CoD. ACL, anterior cruciate ligament; CoD, change of direction; RTS, return to sports.

Of the surveyed PTs, 663 (82.5%) cumulatively believe that 50% or more of the patients with ACL injuries would be able to RTS involving change of direction (CoD) without undergoing an ACL-R (Figure 2A).

Regarding the possibility for individuals with ACL injuries to resume sports involving CoD, the PTs’ answers were widely heterogeneous, with as little as 61 (7.5%) of those surveyed believing that no patient with an ACL injury would ever be able to return to cutting sports without ACL-R (Figure 2B).

Among the surveyed OSs, 139 (69%) believe that 50% or more of patients with ACL injuries would be able to RTS without CoD with no need of an ACL-R (Figure 2A). Of them, 88 (44%) indicated that only 10% or less of the patients with ACL injuries could return to cutting sports avoiding ACL-R (Figure 2B).

Regarding the PT/OSs’ interprofessional relationship, 98 (12%) PTs reported to always rely on a trusted OS, 263 (29.5%) reported they do it often, 276 (34.5%) rarely rely on an OS, whereas 193 (24%) never opt for this option.

**Figure 3:**
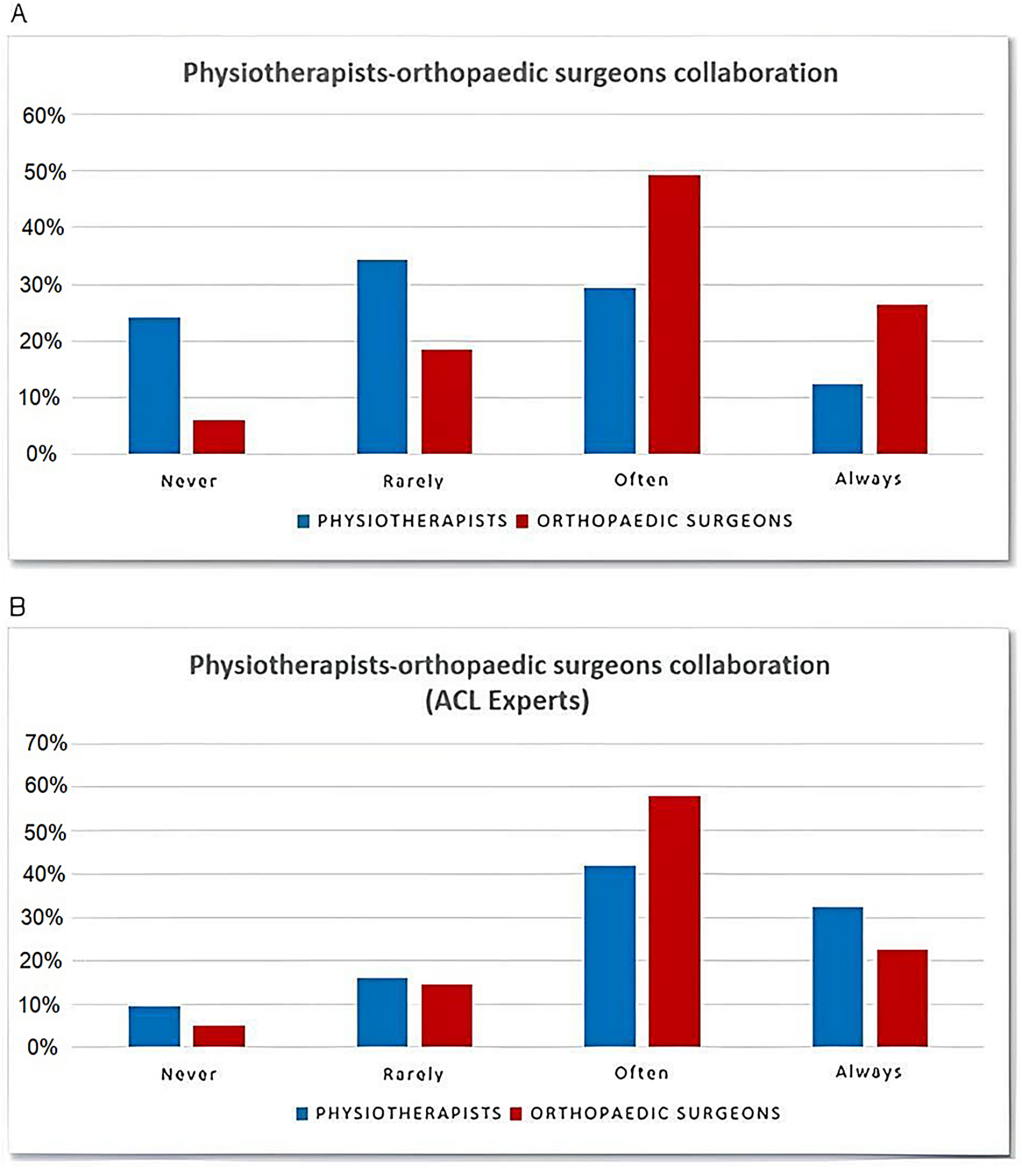
Percentage of professionals cooperating with a trusted PT/OS. **(A)** Percentage of professionals cooperating with a trusted PT/OS. **(B)** Percentage of the ACL-experts cooperating with each other. ACL, anterior cruciate ligament; OS, orthopedic surgeon; PT, physical therapist.

Of the surveyed OSs, 53 (26.5%) indicated they always cooperate with a trusted PT, 90 (49%) answered they often resort to this cooperation, 37 (18.5%) rarely cooperated with a PT, and 12 (6%) never collaborated with a trusted expert in knee rehabilitation (Figure 3A).

### 3.3 Sub-analysis of the surveyed ACL-expert professionals

Filtering the surveyed professionals for their experience in treating ACL lesions, using a cutoff of a minimum of 35 ACL-Rs performed per year for surgeons (20) and the same parameter of annual postoperative rehabilitated ACL cases for PTs, the authors identified a subgroup of 62 OSs and 31 PTs as ‘experts’ in the management of this type of ligamentous lesions.

Among the experienced PTs, 15 (48%) claimed to be sufficiently familiar with the CNCS, while 16 (52%) reported insufficient confidence (less than 60 on a scale of 0–100). A total of 15 (48%) PTs reported to regularly refer to it, whereas 16 (52%) do not use the screening in their daily clinical practice. Only 12 (39%) declared a sufficient confidence screening along their clinical practice, whereas 9 (29%) reported no confidence at all with the CNCS.

In the subgroup of surgeons with high levels of experience in managing ACL lesions, 22 (35%) claimed to have sufficient confidence in the utilization of the CNCS, whereas 40 (65%) responded that their confidence is below 60 on a scale of 0–100. Of the surgeons, 17 (27%) had no confidence whatsoever, 28 (45%) reported that they apply it, and 34 (55%) do not refer to the screening at all. A total of 17 (27%) surgeons indicated that they use the CNCS in their daily clinical practice and hence are sufficiently familiar with it (Figure 1B).

Within the subgroup of the 31 ACL-expert PTs, 25 (80%) declared that, in their opinion, at least half of the patients with ACL injuries would be able to return to non-cutting sports without ACL-R (Figure 2C).

Considering RTS in sports involving CoD, the answers are mainly in the range of 0%–50%. Four PTs (13%) believe that no patients with ACL injuries would ever be able to resume this type of sports without ACL-R (Figure 2D).

Examining the answers of the subgroup of the ACL-expert OSs, 36 of 62 (58%) indicated that at least half of the patients with ACL injuries would be able to return to non-cutting sports with no ACL-R (Figure 2C), whereas 33 of 62 (53%) claimed that only 10% or less of these patients would be able to resume sports involving CoD without ACL-R (Figure 2D).

Regarding the relationship between PTs and OSs, within the subgroup of ACL experts, 10 (32%) PTs indicated they always rely on a trusted OS, 13 (42%) do this often, 5 (16%) do this rarely, and 3 (10%) never opt for such interprofessional cooperation.

As for the subgroup of the ACL-expert OSs, 14 (23%) replied that they always rely on a trusted PT, 36 (58%) often follow this approach, 9 (15%) rarely do this, and 3 (5%) never opt for it (Figure 3B).

Regarding the decision-making process to opt for ACL-R, 13 (42%) of the interviewed ACL-expert PTs reported they are never involved by the OSs in this decision, 10 (32%) reported they are rarely involved, 4 (13%) reported they are often involved, and 4 (13%) reported they are always involved by the surgeon in this decision.

**Figure 4:**
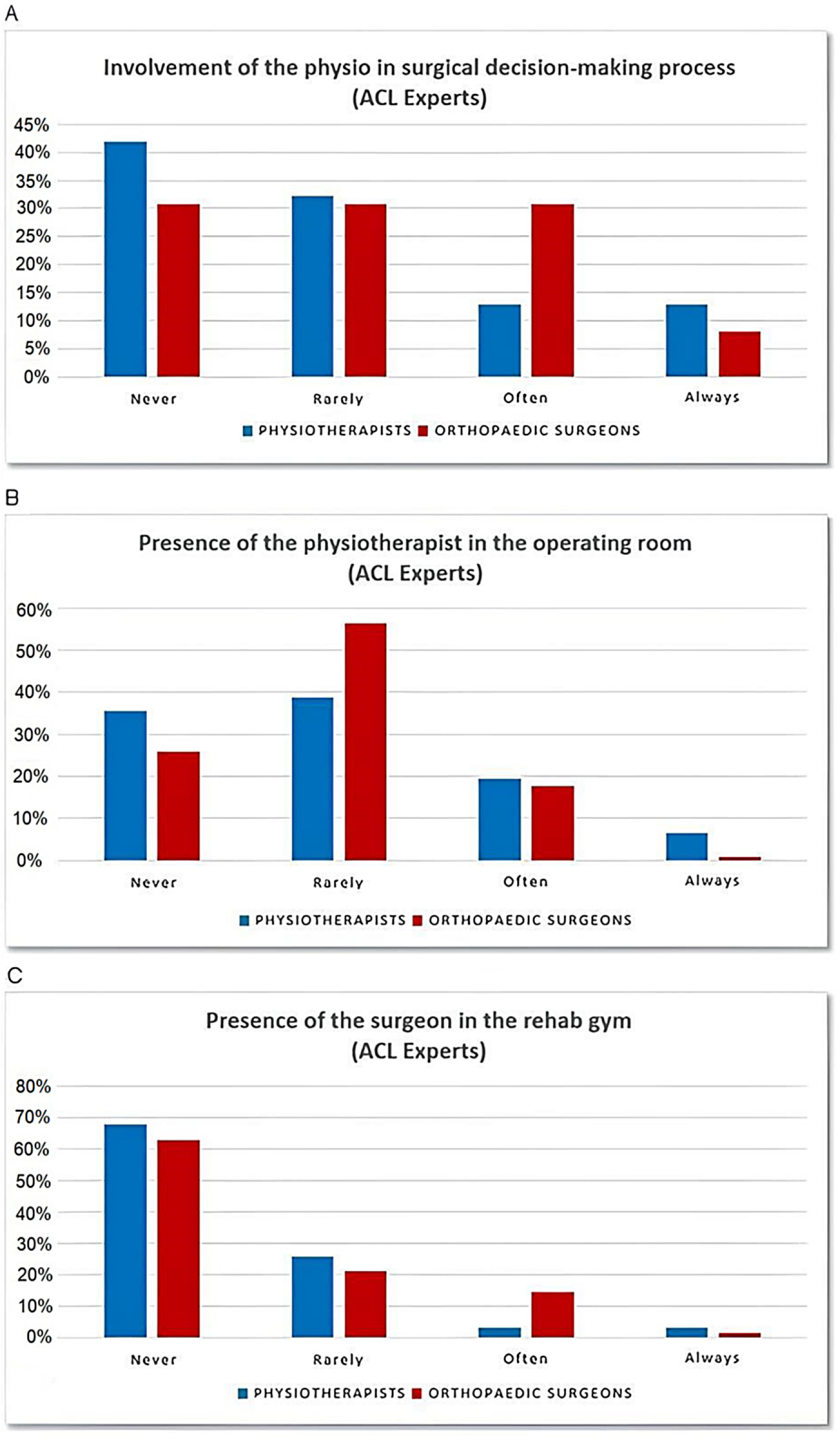
PT-OS collaboration. **(A)** PTs involvement in the ACL-R decision-making process within the ACL experts subgroup. **(B)** Percentage of ACL-expert PTs attending ACL-R in the operating room. **(C)** Percentage of ACL-expert OS attending RTS tests in the rehabilitative setting. ACL, anterior cruciate ligament; ACL-R, anterior cruciate ligament reconstruction; OS, orthopedic surgeon; PT, physical therapist; RTS, return to sports.

Of the surveyed ACL-expert OSs, 19 (31%) indicated that they never involve PTs in their decision to operate on the patient, 19 (31%) rarely involve the rehabilitation professionals, 19 (31%) often consider their opinion, and 5 (8%) always involves the PTs (Figure 4A).

It also emerged that 11 (35.5%) of the ACL-expert PTs have never been in the operating room to attend an ACL-R, 12 (39%) have rarely attended, 6 (19%) often attend, and 2 (6.5%) are always present during their patients’ ACL-Rs.

Of the ACL-expert OSs, 16 (26%) reported to have never been followed by a PT into the operating room during an ACL-R, 35 (56%) reported that this rarely occurs, 11 (18%) reported that it often occurs, and none claimed to be always shadowed by a PT colleague while performing ACL-Rs (Figure 4B).

Of the ACL-expert PTs, 21 68%) have never performed a test for RTS after ACL-R in the presence of an OS, 8 (26%) rarely performed a test for RTS after ACL-R with an OS present, 1 (3%) claimed to do this often, and 1 (3%) always follows this practice. On the other hand, 39 (63%) of the ACL-expert OSs reported they have never attended a RTS testing session for an ACL-R patient, 13 (21%) rarely assist these tests, 9 (14.5%) often attend, and 1 (1.5%) always attends (Figure 4C).

The percentage of ACL patients operated after the application of the screening process was the last aspect investigated by this study. Of the surveyed ACL-expert PTs, 15 (48%) stated that 50% or less of their screened ACL patients underwent ACL-R, whereas only 9 (14.5%) of the ACL-expert OSs reported the same. Analyzing the collected data, it appears that most of the coper/non-coper screened patients eventually opt for ACL-R after the process; yet several surgeries could potential still be avoided.

## 4 Discussion

The findings of the present study highlight a poor level of knowledge, confidence, and implementation in the everyday clinical practice of both PTs and OSs of an initial non-surgical approach for patients with ACL injuries in Italy. The first aspect that emerged is the 4:1 PT:OS ratio within the surveyed population, which well represents the current proportion of clinicians in Italy. In fact, to date there are approximately 66,000 PTs (Atlante Sanità) (21) versus approximately 12,500 OSs (Atlante Sanità) (22) operating in Italy.

Considering the combined subgroup of professionals who are not experts in dealing with ACL injuries, it was found that fewer than 26% of them are familiar with the CNCS and only 20% apply it with sufficient confidence. When examining the opinions of the surveyed professionals regarding the feasibility of RTS without ACL-R, it emerged that there was a certain level of propensity to consider the surgical reconstruction as a relevant factor. In fact, 2%–8% of the professionals combined believe that no player can return to sports with no CoDs without ACL-R, whereas the percentage of PTs and OSs who believe surgery is pivotal in the case of cutting sports increases to 7% and 22%, respectively.

PTs were more optimistic about the possibility of RTS with no ACL-R. This finding supports patients with deficient ACLs patients in pursuing a non-surgical pathway after their injury, as per the published literature (23, 24). Nonetheless, clinicians and patients need to be aware of the limitations of this approach, as highlighted by recently published literature that showed ACL-R as a clinically superior and more cost-effective strategy in comparison with rehabilitation management alone (25).

Of the non-expert professionals who joined the survey, only 12% of PTs and 26% of OSs always rely on a trusted professional counterpart. A quarter of the PTs never rely on a trusted OS, while only 6% of the OSs never collaborate with a PT they trust. Interestingly, the proportions changed when stratifying the data and analyzing the responses collected from ACL experts only, with the percentage of ACL-expert PTs (4%) being considerably lower compared to the percentage of OSs (31%). Furthermore, among the ACL-expert PTs, only 39% reported to apply the screening with confidence, whereas another 29% admitted having no confidence at all with this clinical approach. In addition, the responses show that even when the utilization of the CNCS was indicated, almost one in three ACL-expert PTs would not be able to assist the patient in delivering the appropriate intervention, despite being considered an expert in managing ACL injuries. Such findings highlight the scarcity of the implementation of the CNCS into clinical practice, even among the most experienced ACL rehabilitators.

Among the subgroup of surveyed ACL-expert surgeons, 27% reported to be sufficiently familiar with the application of the screening and a similar percentage reported they were familiar with this practice at all. This implies that some patients are currently denied *a priori* the possibility of avoiding surgery in case of an ACL injury.

As for questions 4 and 5, relating to the possibility of RTS avoiding ACL-R, a remarkable heterogeneity emerged within the answers provided by the PTs, including the subgroup of the ACL experts. Most of them responded that RTS in sports with no CoD is believed to be possible in 70%–100% of cases, whereas the odds decreased to 50% for RTS in cutting sports.

OSs are not as optimistic when asked to indicate the percentage of patients with ACL injuries who would be able to RTS avoiding ACL-R. Their most frequent response was 30% in the case of sports not involving CoD, whereas in the case of cutting sports, their responses fell to 0%–10%.

It should be noted that among the ACL experts, interprofessional collaboration appeared more frequent: a combined percentage of almost 80% of PTs and OSs often or always rely on a trusted counterpart in the management of this type of injury.

ACL-expert PTs claimed to be rarely involved in the surgical decision-making process with patients with ACL injuries. This seems to be confirmed by the ACL-expert OSs; in fact, just over one in three often or always involves the PT in their decision, whereas in 30%–40% of cases, the PT's opinion is never considered.

Considering that the CNCS is a PT-performed screening, involving an ACL rehabilitation expert in the decision-making process, determining whether to manage an ACL injury with an ACL-R or non-surgically, could potentially benefit the patient’s ultimate outcome.

In this context, it would be interesting to investigate whether the choice to not consult the rehabilitator in this important process is linked to a lack of professional liability of the PTs or to reduced confidence by the OSs, or something else entirely.

Regarding the knowledge of other health professional skills, it appears that most of the interviewed PTs rarely attend ACL-R in the operating room, which is confirmed by almost 60% of the OSs. Approximately the same percentage of OSs (60%–70%) reported they do not usually attend RTS test sessions in the rehabilitation setting for their ACL patients.

This could represent a significant limitation in advancing an effective shared clinical practice, as mutual understanding of each other’s areas of expertise and reciprocal physical presence in the respective working environments are part of a patient-centered multidisciplinary process.

Furthermore, increased effective communication could be the key to overcome barriers which, to date, could hinder the implementation of a multidisciplinary approach in a biopsychosocial context in patients with ACL lesions.

According to the scientific literature, the decision to operate on patients with isolated ACL lesions should be abandoned in favor of the CNCS followed by a period of high-quality rehabilitation (13). PTs responded that 30%–50% of the cases could avoid ACL-R after screening, whereas most of the OSs indicated that 70% of patients needed surgery after the screening in any case.

From the overall analysis, more similar responses between the two categories of health professional arise within the ACL-expert subgroups if compared to PTs and OSs who are not routinely treating this type of ligamentous injury.

The collected data showed a marked degree of inconsistency in the ACL non-surgical management. These results demonstrate that the respondent clinicians do not yet possess sufficient familiarity with the CNCS to ensure an adequate level of evidence-based clinical practice for these patients.

The practical aspects that emerged from this study are as follows: (1) the academic and postgraduate training for PTs needs to be more in line with the scientific literature; (2) OSs need to be updated regarding best practice on post-injury management and rehabilitation using a non-surgical approach, carried out by actual ACL-expert PTs; and (3) the utilization of maximizing communicative effectiveness among professional figures could be enhanced by greater sharing of their respective work areas.

### 4.1 Strength and limitations

The structure of the survey, with only 12 questions, and a necessary brief compilation time of 5 min, led to high adherence, ensuring a statistically accurate overview of the current situation in Italy. A limitation of this study was that only 4% of the interviewed PTs deal with a minimum of 35 ACL injuries per year and can therefore be considered ACL experts; therefore, this is not a large enough sub-sample to consider the responses for a statistical analysis. A questionnaire comprising 12 questions without formal validation carries intrinsic limitations for investigating clinical practice in such a complex context as ACL lesions. The use of multiple combined survey distribution channels may have introduced a selection bias; however, given the lack of shared channels between the two groups of health professionals, avoiding this was not feasible.

## 5 Conclusions

This survey identified that a large proportion of the surveyed population of Italian clinicians, especially OSs, placed insufficient trust in the CNCS despite the available literature. The responses collected from PTs, even those highly specialized in dealing with ACL injuries, showed a lack of familiarity and poor application with the screening. ACL-expert PTs and OSs were accustomed to closely cooperating and relying on trusted professional counterparts; however, PTs were often excluded from the decision of whether to take the non-surgical path. Such findings have significant clinical implications, highlighting the lack of application of evidence-based practice in the management of ACL lesions. Further studies should investigate the factors limiting CNCS implementation and how to improve it in clinical practice.

## Data Availability

The original contributions presented in the study are included in the article/Supplementary Material, further inquiries can be directed to the corresponding author.
