# Supplementary material for: Underutilization of coper/non-coper screening in anterior cruciate ligament injuries management in Italy: an online survey
Source: Front Rehabil Sci. 2025 Jan 15;5:1497828. doi: 10.3389/fresc.2024.1497828 (PMC11774934; doi:10.3389/fresc.2024.1497828)
Supplement: Supplementary file 1 [file Datasheet1.pdf]

In this file is reported the entire survey text, translated from the Italian (original language of distribution) to English both for orthopaedic surgeons and physiotherapists.

ORTHOPAEDIC SURGEONS SURVEY

1. What is the average amount (in percentage) of anterior cruciate ligament surgical reconstructions (ACL-R) that you perform every month compared to all the other surgical procedures?

    - 0%
    - 10%
    - 20%
    - 30%
    - 40%
    - 50%
    - 60%
    - 70%
    - 80%
    - 90%
    - 100%

2. What is the approximate number of ACL-R that you perform per year?

    - ____

3. In which cities do you practice as an orthopaedic surgeon?

    - ____

4. In your opinion, what is the percentage of athletes who can return to sports that do NOT involve changes of direction (swimming, running, riding, etc.) without surgical reconstruction (ACL-R), after a total or sub-total ACL injury?

- 0%
- 10%
- 20%
- 30%
- 40%
- 50%
- 60%
- 70%
- 80%
- 90%
- 100%

5. In your opinion, what is the percentage of athletes who can return to sports with changes of direction (football, rugby, basketball, etc.) without surgical reconstruction (ACL-R) after a total or sub-total ACL injury?

- 0%
- 10%
- 20%
- 30%
- 40%
- 50%
- 60%
- 70%
- 80%
- 90%
- 100%

6. How much are you familiar, on a scale between 0 and 100, with the concept of the coper/non-coper screening following an ACL injury?

- o 0%
- o 10%
- o 20%
- o 30%
- o 40%
- o 50%
- o 60%
- o 70%
- o 80%
- o 90%
- o 100%

7. In your daily clinical practice, do you implement a coper/non-coper screening in individuals with isolated ACL injury (note: this is characterized by an initial rehabilitative approach lasting at least 5 weeks before proceeding with an eventual screening that drives the decision-making process between surgical reconstruction Vs non-surgical approach)?

- o YES
- o NO

8. Do you usually involve a physiotherapist in the decision-making process between ACL surgical reconstruction Vs non-surgical approach after an ACL lesion including the outcome of the above-mentioned screening process?

- o NO, never
- o YES, sometimes/rarely
- o YES, frequently/often
- o YES, always

9. Do you normally rely on a trusted physiotherapist or physiotherapy team?

- o NO, never
- o YES, sometimes/rarely
- o YES, frequently/often
- o YES, always

10. What percentage of your patients who have been screened for coper/non-coper usually undergo to ACL-R?

- o 0%
- o 10%
- o 20%
- o 30%
- o 40%
- o 50%
- o 60%
- o 70%
- o 80%
- o 90%
- o 100%

11. Have you ever been followed by a physiotherapist in the operating room during one ACL-R procedure?

- o NO, never
- o YES, sometimes/rarely
- o YES, frequently/often
- o YES, always

12. Have you ever personally attended the coper/non-coper screening tests and/or the RTS tests performed by a physiotherapist?

- NO, never
- YES, sometimes/rarely
- YES, frequently/often
- YES, always

PHYSIOTHERAPISTS SURVEY

1. What is the average amount (in percentage) of anterior cruciate ligament (ACL) injuries that you follow as a physiotherapist every month compared to all the other rehab procedures?

- 0%
- 10%
- 20%
- 30%
- 40%
- 50%
- 60%
- 70%
- 80%
- 90%
- 100%

2. What is the approximate number of ACL injuries that you rehabilitate per year?

- ____

3. In which cities do you practice as a physiotherapist?

- ____

4. In your opinion, what is the percentage of athletes who can return to sports that do NOT involve changes of direction (swimming, running, riding, etc.) without surgical reconstruction (ACL-R), after a total or sub-total ACL injury?

- 0%
- 10%
- 20%
- 30%
- 40%
- 50%
- 60%
- 70%
- 80%
- 90%
- 100%

5. In your opinion, what is the percentage of athletes who can return to sports with changes of direction (football, rugby, basketball, etc.) without surgical reconstruction (ACL-R) after a total or sub-total ACL injury?

- 0%
- 10%
- 20%
- 30%
- 40%
- 50%
- 60%
- 70%
- 80%
- 90%
- 100%

6. How much are you familiar, on a scale between 0 and 100, with the concept of the coper/non-coper screening following an ACL injury?

- 0%
- 10%
- 20%
- 30%
- 40%
- 50%
- 60%
- 70%
- 80%
- 90%
- 100%

7. In your daily clinical practice, do you implement a coper/non-coper screening in individuals with isolated ACL injury (note: this is characterized by an initial rehabilitative approach lasting at least 5 weeks before proceeding with an eventual screening that drives the decision-making process between surgical reconstruction Vs non-surgical approach)?

- YES
- NO

8. Are you usually involved as a physiotherapist in the decision-making process between ACL surgical reconstruction Vs non-surgical approach after an ACL lesion including the outcome of the above-mentioned screening process?

- NO, never
- YES, sometimes/rarely
- YES, frequently/often
- YES, always

9. Do you normally rely on a trusted orthopedic surgeon?

- NO, never
- YES, sometimes/rarely
- YES, frequently/often
- YES, always

10. What percentage of your patients who have been screened for coper/non-coper usually undergo to ACL-R?

- 0%
- 10%
- 20%
- 30%
- 40%
- 50%
- 60%
- 70%
- 80%
- 90%
- 100%

11. Have you ever been in the operating room during one ACL-R procedure?

- NO, never
- YES, sometimes/rarely
- YES, frequently/often
- YES, always

12. Does a surgeon ever personally attend the coper/non-coper screening tests and/or the RTS tests performed by you?

- NO, never
- YES, sometimes/rarely
- YES, frequently/often
- YES, always
